# A case of arrhythmic cardiomyopathy caused by rare multiple gene mutations

**DOI:** 10.3389/fcvm.2025.1598085

**Published:** 2025-07-10

**Authors:** Kaiqin Liang, Hong Wang, Minfang Wu, Meng Cui, Kaiyou Liu, Mintao Gan, Wengong Cheng, Aiqiong Huang

**Affiliations:** ^1^ Department of Internal Medicine Nursing, Nursing College of Guangxi Medical University, Nanning, Guangxi, China; ^2^ Department of Cardiology, The People's Hospital of Guangxi Zhuang Autonomous Region, Nanning, Guangxi, China; ^3^ Department of Clinical Medicine, Youjiang University of Ethnic Medicine, Baise, Guangxi, China; ^4^ Department of Radiology, The People's Hospital of Guangxi Zhuang Autonomous Region, Nanning, Guangxi, China; ^5^ Department of Clinical Medicine, Dalian Medical University, Dalian, Liaoning, China; ^6^ Department of Cardiology, Nanyang Second People's Hospital, Nanyang, Henan, China; ^7^ School of Foreign Languages, Guangxi Medical University, Nanning, Guangxi, China

**Keywords:** arrhythmogenic cardiomyopathy, sudden cardiac death, cardiovascular magnetic resonance, desmoplakin, phenotype, pedigree

## Abstract

Arrhythmogenic cardiomyopathy (ACM) is an inherited cardiomyopathy characterized by a high risk of ventricular tachycardia and sudden cardiac death, often involving the right ventricle or both ventricles, with the initial onset usually in adolescence or young adulthood, and most cases can be diagnosed before the age of 40 years. Studies have shown that ACM is often caused by mutations in genes encoding desmosomal proteins, with a small proportion caused by mutations in nonencoding desmosomal proteins. In this paper, we report a patient with biventricular arrhythmogenic cardiomyopathy who presented with recurrent syncope, paroxysmal ventricular tachycardia, and heart failure in old age. Genetic testing revealed that the patient (proband) carried three rare genetic variants in the genes encoding desmosomal and nondesmosomal proteins at the same time. No relevant reports were found in the literature review, and the phenotypic penetrance age differences among family members carrying the same genetic variant were also large, further indicating the complexity of ACM genotype–phenotype expression. We treated the family members for one year of follow-up. To provide more references for risk assessment, individualized management and genetic counselling of ACM patients are needed.

## Introduction

The main pathophysiological change in ACM is that the normal ventricular myocardium is gradually replaced by fibres or fibroadipose tissue, and this pathological process slows the conduction of intraventricular electrical signals, triggers fatal ventricular arrhythmias through scar-related mechanisms (1, 2), and gradually results in heart failure. Because right ventricular involvement is relatively common, it was previously known as arrhythmogenic right ventricular cardiomyopathy (ARVC). In recent years, 56% of patients have bilateral ventricular involvement, and a small proportion of patients present with left ventricular involvement (3, 4). Therefore, the International Task Force (ITF) proposed the definition of ACM in 2010 and classified ACM into arrhythmogenic right ventricular cardiomyopathy (ARVC), arrhythmogenic left ventricular cardiomyopathy (ALVC) and arrhythmogenic biventricular cardiomyopathy (biventricular cardiomyopathy), which are currently diagnosed and evaluated in terms of cardiac structural imaging, histology, electrocardiogram, arrhythmia characteristics, and genetic testing via the 2020 “Padua cardiomyopathy” (5, 6). Previous studies have shown that ACM is an important cause of sudden death in young people and athletes and has a significant familial genetic predisposition, with an incidence between 1:1,000 and 1:2,000, and causative mutations can be found in approximately half of patients. The Clinical Genome Resource (ClinGen) identifies genetic variants such as DES, DSC2, DSG2, DSP, JUP, PKP2, and TMEM43 as clearly associated with ACM pathogenesis (7, 8). In this case, the proband presented with typical manifestations of ACM, such as syncope, paroxysmal ventricular tachycardia, and heart failure, and the multimodality imaging results revealed biventricular involvement. We performed genetic testing, comprehensive evaluation, treatment, and follow-up for the patient (proband) and his family members, as reported below.

## Case presentation

**Figure 1:**
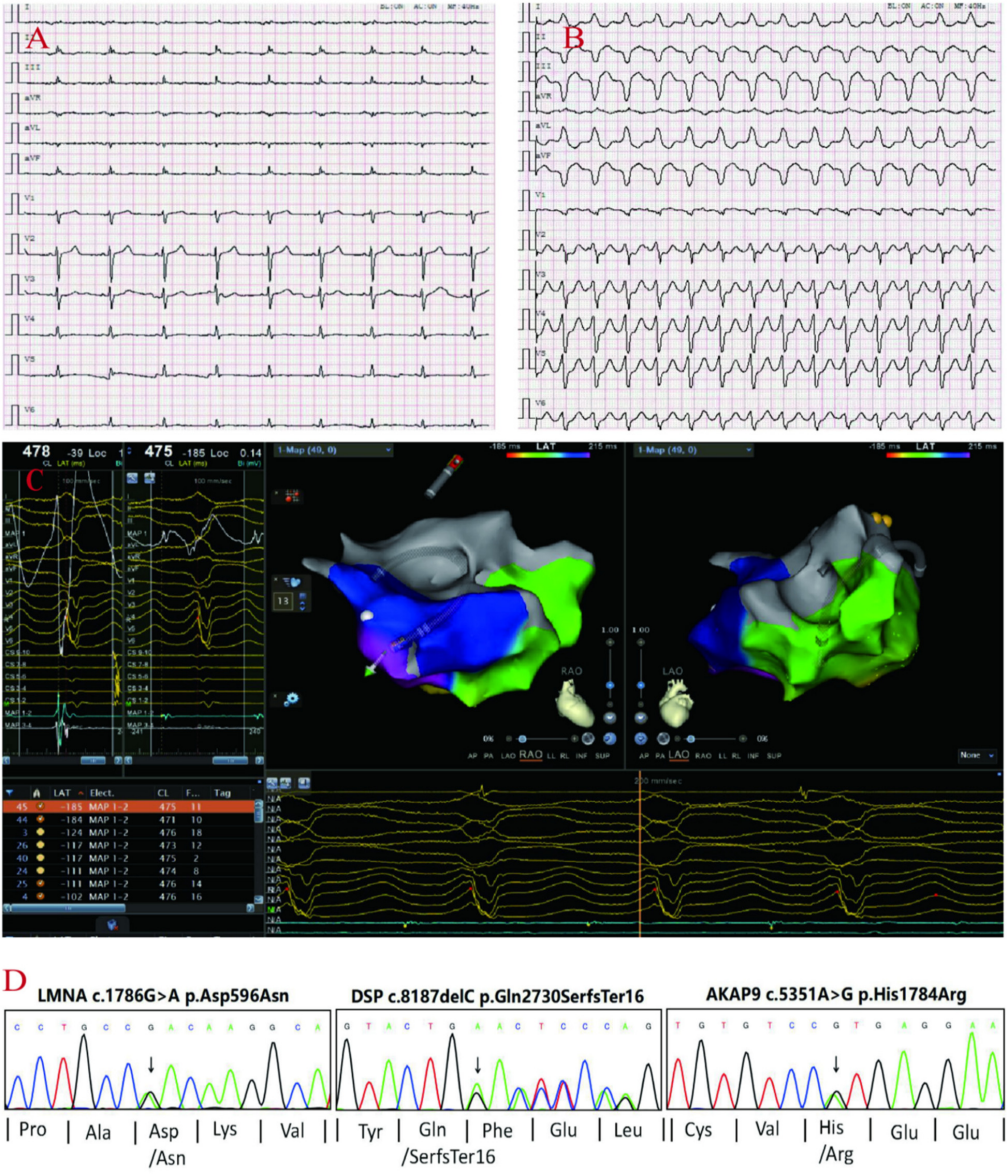
proband's ECG: Q-T interval prolongation, low voltage of QRS complex in limb leads **(A)**, non-persistent ventricular tachycardia (QRS group with left bundle branch block pattern) **(B)**. Electrophysiological: the earliest V wave measured in the chamber speed state **(C)**. Sanger sequencing confirmed the the variants in DSP, AKAP9, LMNA genes in the proband **(D)**.

**Figure 2:**
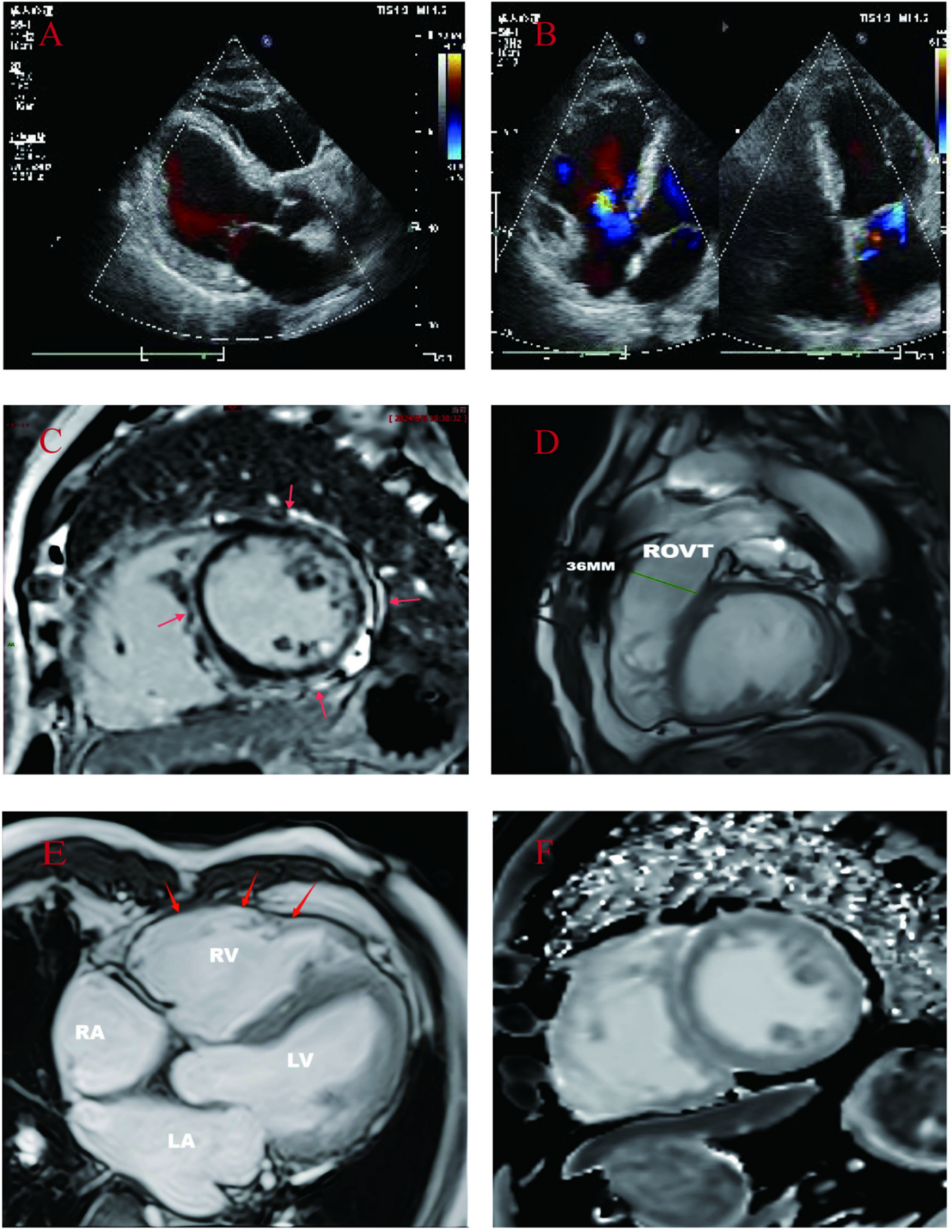
**(A,B)**: Proband's echocardiogram: showed whole heart enlargement, overall movement weakened, left ventricular EF45% FS23%. **(C–F)**: CMR images of probands. **(C)**: Cardiac short-axis LGE: delayed enhancement between the free wall of the middle left ventricle and the subepicardium of the right ventricle, and delayed enhancement between the muscular walls of the interventricular septum. **(D)**: Right ventricular outflow tract widening: 36 mm. **(E)**: Left and right ventricular wall multiple thinning, right ventricular wall tumor (local myocardial contradictory movement). **(F)**: Short axis native T1: increased T1 value between subepicardial left ventricular free wall and interventricular septum muscle wall.

A 71-year-old male patient (proband) presented to the hospital with recurrent sudden syncope, and comprehensive evaluation revealed that his electrocardiogram revealed a prolonged Q-T interval, low-voltage QRS complexes in the limb leads, and multiple episodes of nonsustained ventricular tachycardia (QRS complexes with left bundle branch block morphology) (Figures 1A–C); echocardiography and magnetic resonance imaging revealed global enlargement of the left and right ventricles, global hypokinesia, multiple thinning of the ventricular wall, ventricular aneurysm in the right ventricle (regional myocardial paradoxical movement), widening of the right ventricular outflow tract, and decreased left and right ventricular ejection fractions (Figures 2A,B,D,E); magnetic resonance enhancement (LGE) revealed subepicardial free walls in the middle left ventricle, interventricular septal wall, and transmural myocardial fibrosis images in the right ventricular inferior wall (Figures 2C,F) (Supplementary Material S1), and coronary angiography revealed plaque formation in the proximal left anterior descending artery, 30% stenosis, and no significant stenosis in the remaining vessels. In view of the proband's clinical manifestations and multimodality imaging results, we suspected that he had ACM and performed whole exome sequencing (WES): DNA was first extracted from peripheral blood, libraries were constructed using Agilent SureSelectXT V6, and sequenced on the Illumina NovaSeq 6,000 platform.Data analysis was screened by BWA alignment (GRCh37/hg19), PICARD de-duplication, SnpEff and ANNOVAR annotation, focusing on exonic regions and non-coding variants affecting splicing (Splice-AI > 0.2), combined with disease database and Shengxin software prediction. The pathogenicity of genetic variants was judged according to the American College of Medical Genetics and Genomics (ACMG) and Association for Molecular Pathology (AMP) criteria (9), the results revealed that he carried three rare mutations: the heterozygous missense variant c.1786G > A (p.Asp596Asn) in the *LMNA* gene(suspected causative), the heterozygous variant c.8187delC (p.Gln2730SerfsTer16) in the *DSP* gene(suspected causative), and the heterozygous missense variant c.5351A > G (p.His1784Arg) in the *AKAP9* gene (unknown significance) (Figure 1D). All variants were verified by Sanger sequencing, and co-segregation analysis was performed on family members. The proband was diagnosed with arrhythmogenic biventricular cardiomyopathy and Q-T interval prolongation according to the revised 2020 ITF diagnostic criteria for ACM and was considered to have a high risk of malignant arrhythmia and sudden death. Radiofrequency ablation and drug therapy were performed after consultation with the proband and his family members, considering the hereditary nature of the disease, we conducted genetic testing and comprehensive evaluations for the proband's family members.

**Figure 3:**
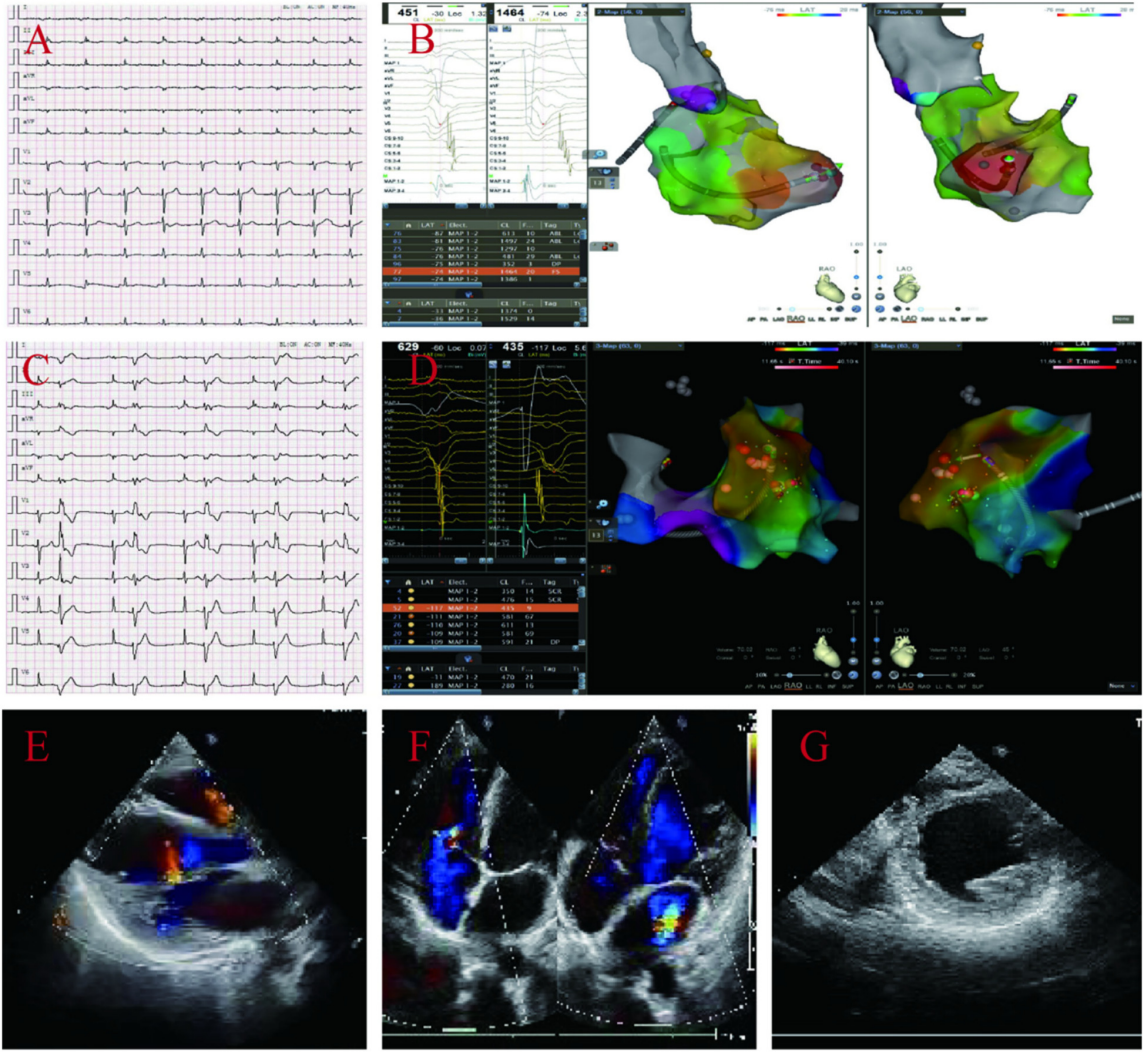
ECG and electrophysiological of the proband's daughter A display: frequent multi-source ventricular premature beats, low limb lead voltage **(A–D)**. Echocardiography: enlarged left atrial ventricle, overall weakened ventricular wall motion, reduced left ventricular systolic and diastolic function, and no abnormalities in the right atrium and ventricle, left ventricular EF44% and FS22% **(E–G)**.

The proband's daughter A (39 years old) also presented with multiple syncope symptoms, and electrocardiogram and electrophysiology revealed frequent multifocal ventricular premature beats and low voltage in limb leads; echocardiography revealed left atrial ventricular enlargement, global weakening of wall motion, decreased left ventricular systolic and diastolic function, and no abnormalities in the right atrium and ventricle (Figures 3A–G); multiple attempts to perform MRI for her failed to complete the examination because she developed claustrophobic syndrome manifestations such as fear, tachycardia, and increased respiration during the examination. Genetic testing revealed that she carried two rare mutations: the heterozygous variant c.8187delC (p.Gln2730SerfsTer16) in the *DSP* gene(suspected causative), and the heterozygous missense variant c.5351A > G (p.His1784Arg) in the *AKAP9* gene (unknown significance)(Supplementary Material S3) which was diagnosed as ALVC based on the above examination results, and comprehensive treatment, such as radiofrequency ablation and drugs, was given.

The proband's daughter B (37 years old), genetic testing revealed that she carried the same genetic mutation as the proband did, but she currently had no obvious palpitation symptoms and had not developed syncope. Long-range electrocardiogram revealed ventricular premature beats of 224 beats/24 h, no other arrhythmias were found, and cardiac ultrasound revealed no cardiomegaly or ventricular wall abnormalities.

**Figure 4:**
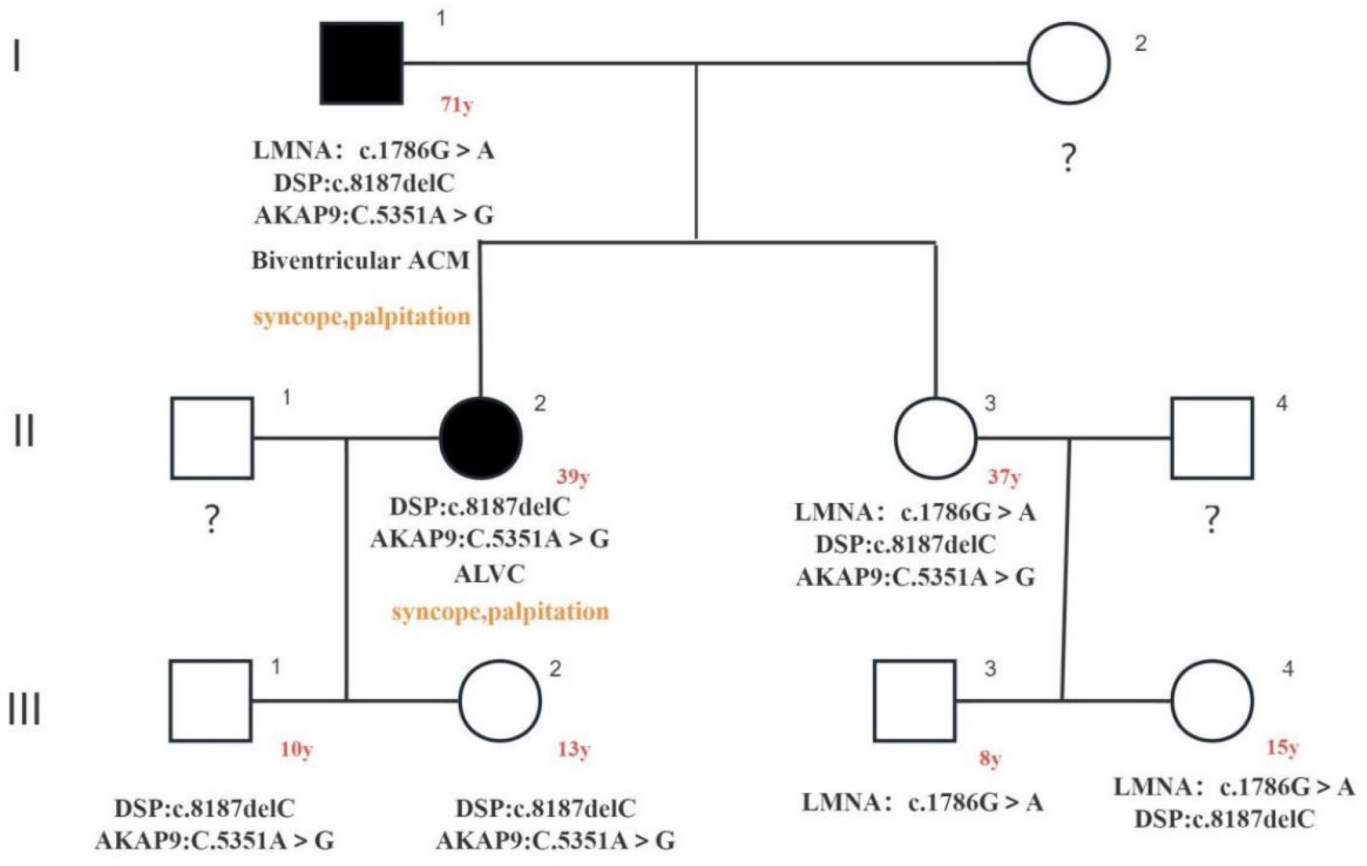
The image illustrates family pedigree of three rare gene variants: c.1786G > A (LMNA: p.Asp596Asn het) heterozygous missense variant in the LMNA gene and c. in the DSP gene8187delC (DSP: p.Gln2730SerfsTer16 het) heterozygous variant, both of which were identified as suspected causative variants; AKAP9 gene c.5351A > G (AKAP9: p. His1784Arg het) heterozygous missense variant, which was identified as a variant of uncertain significance. Squares indicated male family members and circles denoted female family members. The arrow indicates the index proband. Solid black symbols in the pedigree denote individuals with a history of cardiac disease (ACM). The proband's phenotype was biventricular ACM, and the proband's daughter A (39-year-old) exhibited an ALVC phenotype. Both individuals experienced syncope episodes. No phenotypic expression or incomplete penetrance has been observed in the proband's daughter B (37-year-old) and his other offspring.

ACM has previously been shown to be an important cause of sudden cardiac death in young people, especially athletes (10). Early recognition is important. Therefore, we expanded the scope of genetic testing of family members and found that all four grandchildren of the proband carried suspected causative gene variants (Supplementary Material S3) and four grandchildren were assessed to have not yet developed symptoms such as palpitations and syncope (Figure 4, Table 1).

**Table 1:** Adult characteristics in pedigrees (before the follow-up).

Characteristics	Family Members		
	Proband	Proband's daughter A	Proband's daughter B
	(Male, 71 years)	(39 years)	(37 years)
Symptoms	Syncope	Syncope	None
	PSVT	PSVT	
	Biventricular reduced ejection fraction	Reduced LVEF	
Phenotype	Biventricular ACM, Combined with Q-T interval prolongation	ALVC	Non-penetrant or incomplete penetrance
Rare genetic variants	LMNA:c.1786G > A	DSP:c.8187delC	LMNA:c.1786G > A
	DSP:c.8187delC	AKAP9:c.5351A > G	DSP:c.8187delC
	AKAP9:c.5351A > G		AKAP9:c.5351A > G
Other comorbidities	None	None	None
Medication History	None	None	None
Sleep quality	Normal	Normal	Normal
Exercise	Agriculture labor	Rarely	Rarely
Smoking Status	∼20 cigarettes/day for 55 years	No	No
Diet	High-fat and high-protein foods	Asian diet (low in salt, normal protein and fat content)	Asian diet (low in salt, normal protein and fat content)
Drinking Status	Consume alcohol regularly (38%–50% vol. 3–4 times/week, 250–500 ml/ time)	Seldom consume alcohol	Seldom consume alcohol
Vocation	Farmer (Traditional farming)	Electronic parts factory workers (Working hours: 8–9 h/day)	Grocery clerk (Working hours: 8–9 h/day)
Stressful Events	Lose money	Childbirth	Childbirth
BMI	22	20.8	23.4

We used a combination of outpatient follow-up and telephone follow-up to follow up with family members according to individual risk stratification from the aspects of drug supervision, lifestyle adjustment, monitoring of related disease symptoms and arrhythmia, and reexamination of cardiac structure and function (8, 11). The current follow-up results revealed that the proband and his daughter A could take the drug on time after radiofrequency ablation and adopt a scientific lifestyle without syncope or obvious palpitation symptoms. Regular reexamination via cardiac B ultrasound revealed no progressive enlargement of the heart and no deterioration of cardiac function, and long-term ECG revealed no frequent ventricular premature beats or ventricular tachycardia. The proband's daughter B and the proband's grandchildren have thus far had no significant associated symptoms, and family members have been able to perform exercise restrictions. We will continue to follow family members and actively take scientific measures to prevent and treat cardiac malignant events such as sudden cardiac death.

## Discussion

Previous studies have identified mutated genes that are closely associated with ACM pathogenesis, including genes encoding desmosomal proteins (PKP2, DSP, DSG2, DSC2, JUP) (7, 12–14). Desmosomes are key proteins of intercellular junctions and are abundantly expressed in tissues subjected to mechanical compressive stresses, such as the epidermis and myocardium, and their important function is to mechanically connect adjacent cells by connecting intermediate filaments of adjacent cells to form a unified cytoskeletal network (15). When desmosomal protein function is deficient, mechanical stress causes cells to detach from cells, resulting in cell death, which in turn produces local inflammation and is eventually replaced by fibres or fibroblasts. DSP encodes desmosomal protein, which is the most abundant component in desmosomes (16), Previous studies have shown that DSP mutation carriers predominantly exhibit left ventricular involvement and are prone to left ventricular dysfunction (manifested as reduced left ventricular ejection fraction, LVEF). Approximately one-third of patients display late gadolinium enhancement in the left ventricle while maintaining normal left ventricular systolic function. These cases more frequently present with left ventricular-originated ventricular arrhythmias, and an LVEF <55% is significantly associated with severe ventricular arrhythmias in DSP-related cases. Additionally, DSP mutation carriers demonstrate the highest rate of sudden cardiac death among arrhythmogenic cardiomyopathy (ACM) cases, accounting for about 11% of total sudden deaths caused by desmosomal gene mutations. DSP gene-related cardiomyopathy progresses rapidly and has a poor prognosis (17–20). A heterozygous DSP: c.8187dleC mutation was detected in the proband's pedigree, and a query of the population frequency database identified this variant as a rare variant (1,000 Genomes: None, ESP6500: None, ExAC: None), which changes polar uncharged glutamine at position 2,730 to polar uncharged serine, followed by protein frameshift expression, and a stop codon at amino acid 15 thereafter, thus affecting desmosomal protein expression. In this case, the proband and his two daughters carried the same heterozygous mutation of DSP: c.8187dleC (DSP: p.Gln2730SerfsTer16 het) in the family, and the proband phenotypically presented with arrhythmogenic biventricular cardiomyopathy and has now presented with global heart failure. His daughter A (39 years old) was phenotypically ALVC, and echocardiography revealed decreased left ventricular contractility, which is consistent with previous literature reports of susceptibility to left ventricular dysfunction carrying DSP gene mutations. The proband's daughter B (37 years old) was not found to have arrhythmia and structural and functional changes in the heart at present, considering that the current phenotype was not penetrant or that the possibility of incomplete penetrance was high. The proband had recurrent ventricular tachycardia with a left bundle branch block morphology in the QRS complex originating from the right ventricular base, and frequent multifocal ventricular premature beats originating from the left ventricular free wall and right ventricular outflow tract were observed in the proband's daughter A through electrophysiological examination. In an observational study of 74 patients with ACM, Eva Cabrera-Borrego reported that sustained monomorphic ventricular tachycardia (SMVT) occurring in those carrying *DSP* mutations presented as a right bundle branch block morphology originating from the inferolateral segment of the left ventricle (21).

In recent years, an increasing number of ACM cases associated with non-desmosomal gene mutations have been discovered. These mutations are present in genes encoding proteins such as intermuscular line protein (*DES*), lamin A/C antigen (*LMNA*), transmembrane protein 43 (*TMEM43*), titin (*TTN*), and filament protein C (*FLNC*) (22); the proteins encoded by these genes have multiple biological functions, including cardiomyocyte cytoskeletal structure, calcium handling, sodium transport, and cytokine signalling (23, 24). *LMNA* encodes lamin A/C, which belongs to the lamin family. Lamin A/C is involved in DNA replication, chromatin structure, spatial localization of nuclear pore complexes, binding of nuclear membrane proteins, and nuclear stabilization. In this case, the family carried a heterozygous missense variant c.1786G > A (p.Asp596Asn) in the *LMNA* gene, which was a rare variant according to population frequency databases. This variant changes the amino acid from a polar, negatively charged aspartate to a polar, uncharged asparagine, affecting protein function. Eva Cabrera-Borrego reported that ACM patients carrying *LMNA* gene mutations had the highest recurrence rate of ventricular tachycardia in their study (21); therefore, continued close follow-up of the proband is needed. The proband's daughter B (37 years old) also carried the c.1786G > A heterozygous missense variant, and no significant manifestations of cardiomyopathy have been identified in this patient. However, previous studies have shown that cardiomyopathy patients carrying a causative mutation in the *LMNA* gene have a wide range of onset ages and can develop the disease any time from adolescence to old age, with an average age of 40 years; penetrance increases with age, with 50% penetrance in autosomal dominant patients aged 30–40 years and 90% penetrance in patients aged >40 years (25). Therefore, although the proband's daughter B is currently asymptomatic, regular follow-up should be performed.

The AKAP9 gene encodes a protein that belongs to a-kinase anchoring protein, which functions by binding to protein kinase a (PKA) regulatory subunit and restricting the holoenzyme to discrete positions in the cell and can also participate in mediating the protein kinase A pathway and participating in the phosphorylation of L-type calcium channels and potassium channels in cardiomyocytes. Previous studies have shown that AKAP9 gene mutations are associated with the development of autosomal dominant long QT syndrome (26). In this case, both the proband and his two daughters carried the rare AKAP9 gene c.5351A > G heterozygous missense variant (AKAP9: p.His1784Arg het), which was found to be a rare variant by querying the population frequency database, but multiple bioinformatics prediction software programs (including SIFT and Polyphen-2, etc.) cross-predicted that the results were mostly harmless, suggesting that amino acid changes caused by this variant may not have an effect on protein function. However, the ECG of the proband in this case showed prolonged Q-T interval (QTc = 476 ms), Although his two daughters carried the same heterozygous missense variant in the AKAP9 gene (c.5351A > G, AKAP9:p.His1784Arg het) as their father, repeated ECG examinations revealed no Q-T interval prolongation. Given the proband's age and unhealthy lifestyle, his prolonged Q-T interval may have been acquired rather than congenital, with little association to the AKAP9 c.5351A > G heterozygous missense variant (AKAP9:p.His1784Arg het). However, previous literature reports that individuals with a QTc interval >440 ms have a 2–3 times higher risk of sudden cardiac death compared to those with a QTc interval <440 ms (27). The proband carries suspected pathogenic mutations in both the LMNA and DSP genes, along with QTc prolongation, indicating a poor prognosis and high risk of sudden death. Active preventive measures should therefore be taken.

In summary, three rare gene mutations were involved in the pedigree of this proband: the c.1786G > a heterozygous missense variant in the LMNA gene, the c.8187delC heterozygous variant in the DSP gene, and the c.5351A > G heterozygous missense variant in the AKAP9 gene. The proband also carried the above three variants, namely, phenotypically arrhythmogenic biventricular cardiomyopathy with a prolonged Q-T interval and recurrent ventricular tachycardia leading to syncope with global heart failure, and was severely ill, which may be associated with the additive effects of carrying two suspected causative gene variants, advanced age, and unhealthy lifestyle. The proband's daughter A (39 years old) also carried the c.8187delC heterozygous variant of the DSP gene and the c.5351A > G heterozygous missense variant of the AKAP9 gene with the ALVC phenotype and no Q-T interval prolongation and has now experienced decreased left ventricular contractility and has experienced syncope, with multifocal ventricular premature beats monitored by electrocardiogram and electrophysiological examination. Syncope has previously been associated with adverse outcomes in patients with ARVC, including sudden cardiac death (28). Although the proband's daughter A carried the same heterozygous variant of the suspected causative gene DSP gene c.8187delC as the proband did, the phenotypic penetrance occurred significantly earlier than that of the proband did, and no influencing factors, such as comorbidities, unhealthy lifestyles, drugs and toxic and hazardous substances, were found after evaluation. The differences in the phenotypic penetrance age between the father and his daughter A may be related to genetic modifiers, polygenic background differences and other factors.

The proband's daughter B (37 years old) carried the same three genetic variants as the proband did, and the follow-up results from the past year revealed that she still had no obvious symptoms, that no cardiac structural or functional abnormalities were detected via echocardiography, and that no arrhythmias were detected via long-range electrocardiography. Taken together, the findings of previous studies suggest that the proband's daughter B has no or incomplete phenotype at present, which may be related to genotype-specific mechanisms: the same variant may produce different amino acid sequences due to alternative splicing or protein modification differences, and the corresponding protein expression is different.

The proband had four grandchildren with a current age of 8–15 years. The results of genetic testing revealed that they all inherited the suspected causative gene from their mothers, and the follow-up results from the past year revealed that the children performed exercise restrictions according to the doctor's advice and could adhere to a scientific lifestyle. No ACM-related manifestations occurred after evaluation, and we will continue to follow up with the family members.

## Conclusion

In this case, the proband also carried three rare genetic variants: the c.1786G > a heterozygous missense variant of the LMNA gene, the c.8187delC heterozygous variant of the DSP gene, and the c.5351A > G heterozygous missense variant of the AKAP9 gene. No relevant reports were found in the literature review, which may be the first discovery. The proband's phenotype was biventricular affected ACM, the phenotype was penetrant in later life, the condition was severe, and there were unhealthy lifestyles such as smoking, alcohol consumption, and a high-fat and high-protein diet; the proband's daughter A (39 years old) and proband carried the same heterozygous variant of the suspected pathogenic DSP gene c.8187delC, the phenotype was ALVC, and there were no complications or unhealthy lifestyles, but the age of phenotypic penetrance was significantly earlier than that of the proband (her father); the proband's daughter B (37 years old) carried the same three rare gene variants as the proband (her father), there were no complications or unhealthy lifestyles, and the phenotype has not yet been penetrant or incomplete. The pedigree characteristics of this case further suggest the complexity of the aetiology and pathogenesis of ACM, and pathogenic genes and environmental factors may be involved in phenotypic expression. We combined the clinical manifestations, phenotypes, pathogenic genes, environmental factors (lifestyle and behavioural habits, etc.), and age characteristics of family members to risk stratify the disease, administer individualized follow-up management, and actively prevent and treat adverse events such as sudden cardiac death; at the same time, it is recommended that family members perform genetic counselling when fertility needs to effectively prevent the occurrence of genetic diseases.

## Data Availability

The original contributions presented in the study are included in the article/Supplementary Material, further inquiries can be directed to the corresponding author.

## References

[1] Nava A Thiene G Canciani B Scognamiglio R Daliento L Buja G Familial occurrence of right ventricular dysplasia: a study involving nine families. J Am Coll Cardiol. (1988) 12(5):1222–8. 10.1016/0735-1097(88)92603-4 3170963

[2] Corrado D Anastasakis A Basso C Bauce B Blomström-Lundqvist C Bucciarelli-Ducci C Proposed diagnostic criteria for arrhythmogenic cardiomyopathy: European task force consensus report. Int J Cardiol. (2024) 395:131447. 10.1016/j.ijcard.2023.131447 37844667

[3] Romero J Mejia-Lopez E Manrique C Lucariello R. Arrhythmogenic right ventricular cardiomyopathy (ARVC/D): a systematic literature review. Clin Med Insights Cardiol. (2013) 7:97–114. 10.4137/CMC.S10940 23761986 PMC3667685

[4] Miles C Finocchiaro G Papadakis M Gray B Westaby J Ensam B Sudden death and left ventricular involvement in arrhythmogenic cardiomyopathy. Circulation. (2019) 139(15):1786–97. 10.1161/CIRCULATIONAHA.118.037230 30700137 PMC6467560

[5] Corrado D Zorzi A Cipriani A Bauce B Bariani R Beffagna G Evolving diagnostic criteria for arrhythmogenic cardiomyopathy. J Am Heart Assoc. (2021) 10(18):e021987. 10.1161/JAHA.121.021987 34533054 PMC8649536

[6] Graziano F Zorzi A Cipriani A De Lazzari M Bauce B Rigato I The 2020 “Padua criteria” for diagnosis and phenotype characterization of arrhythmogenic cardiomyopathy in clinical practice. J Clin Med. (2022) 11(1):279. 10.3390/jcm11010279 35012021 PMC8746198

[7] Arbelo E Protonotarios A Gimeno JR Arbustini E Barriales-Villa R Basso C 2023 ESC guidelines for the management of cardiomyopathies. Eur Heart J. (2023) 44(37):3503–626. 10.1093/eurheartj/ehad194 37622657

[8] Gerull B Brodehl A. Insights into genetics and pathophysiology of arrhythmogenic cardiomyopathy. Curr Heart Fail Rep. (2021) 18:378–90. 10.1007/s11897-021-00532-z 34478111 PMC8616880

[9] Richards CS Bale S Bellissimo DB Das S Grody WW Hegde MR ACMG recommendations for standards for interpretation and reporting of sequence variations: revisions 2007. Genet Med. (2008) 10(4):294–300. 10.1097/GIM.0b013e31816b5cae 18414213

[10] Corrado D Link MS Calkins H. Arrhythmogenic right ventricular cardiomyopathy. N Engl J Med. (2017) 376:61–72. 10.1056/NEJMra1509267 28052233

[11] Calkins H Corrado D Marcus F. Risk stratification in arrhythmogenic right Ventricular cardiomyopathy. Circulation. (2017) 136(21):2068–82. 10.1161/CIRCULATIONAHA.117.030792 29158215 PMC5777304

[12] Ye JZ Delmar M Lundby A Olesen MS. Reevaluation of genetic variants previously associated with arrhythmogenic right ventricular cardiomyopathy integrating population-based cohorts and proteomics data. Clin Genet. (2019) 96(6):506–14. 10.1111/cge.13621 31402444

[13] Mundisugih J Ravindran D Kizana E. Exploring the therapeutic potential of gene therapy in arrhythmogenic right ventricular cardiomyopathy. Biomedicines. (2024) 12(6):1351. 10.3390/biomedicines12061351 38927558 PMC11201581

[14] Ackerman MJ Priori SG Willems S Berul C Brugada R Calkins H Heart rhythm society (HRS); European heart rhythm association (EHRA). HRS/EHRA expert consensus statement on the state of genetic testing for the channelopathies and cardiomyopathies: this document was developed as a partnership between the heart rhythm society (HRS) and the European heart rhythm association (EHRA). Europace. (2011) 13(8):1077–109. Erratum in: Europace. 2012 Feb;14(2):277. Probst, Vincent [added]; Schwartz, Peter J [added]; Kääb, Stefan [added]; Kirchhof, Paulus [added]. 10.1093/europace/eur245 21810866

[15] Vermij SH Abriel H van Veen TA. Refining the molecular organization of the cardiac intercalated disc. Cardiovasc Res. (2017) 113(3):259–75. 10.1093/cvr/cvw259 28069669

[16] Rampazzo A Nava A Malacrida S Beffagna G Bauce B Rossi V Mutation in human desmoplakin domain binding to plakoglobin causes a dominant form of arrhythmogenic right ventricular cardiomyopathy. Am J Hum Genet. (2002) 71(5):1200–6. 10.1086/344208 12373648 PMC385098

[17] Smith ED Lakdawala NK Papoutsidakis N Aubert G Mazzanti A McCanta AC Desmoplakin cardiomyopathy, a fibrotic and inflammatory form of cardiomyopathy distinct from typical dilated or arrhythmogenic right ventricular cardiomyopathy. Circulation. (2020) 141(23):1872–84. 10.1161/CIRCULATIONAHA.119.044934 32372669 PMC7286080

[18] Gasperetti A James CA Carrick RT Protonotarios A te Riele ASJM Cadrin-Tourigny J Arrhythmic risk stratification in arrhythmogenic right ventricular cardiomyopathy. Europace. (2023) 25(11):euad312. 10.1093/europace/euad312 37935403 PMC10674106

[19] Di Lorenzo F Marchionni E Ferradini V Latini A Pezzoli L Martino A DSP-related cardiomyopathy as a distinct clinical entity? Emerging evidence from an Italian cohort. Int J Mol Sci. (2023) 24(3):2490. 10.3390/ijms24032490 36768812 PMC9916412

[20] Towbin JA McKenna WJ Abrams DJ Ackerman MJ Calkins H Darrieux FCC 2019 HRS expert consensus statement on evaluation, risk stratification, and management of arrhythmogenic cardiomyopathy. Heart Rhythm. (2019) 16(11):e301–72. 10.1016/j.hrthm.2019.05.007 31078652

[21] Cabrera-Borrego E Bermúdez-Jiménez FJ Gasperetti A Tandri H Sánchez-Millán PJ Molina-Lerma M Electrophysiological phenotype-genotype study of sustained monomorphic ventricular tachycardia in inherited, high arrhythmic risk, left ventricular cardiomyopathy. Circ Arrhythm Electrophysiol. (2024) 17(12):e013145. 10.1161/CIRCEP.124.013145 39611258

[22] Bermúdez-Jiménez FJ Carriel V Brodehl A Alaminos M Campos A Schirmer I Novel desmin mutation p.Glu401Asp impairs filament formation, disrupts cell membrane integrity, and causes severe arrhythmogenic left ventricular cardiomyopathy/dysplasia. Circulation. (2018) 137(15):1595–610. 10.1161/CIRCULATIONAHA.117.028719 29212896

[23] Forleo C Carmosino M Resta N Rampazzo A Valecce R Sorrentino S Clinical and functional characterization of a novel mutation in lamin a/c gene in a multigenerational family with arrhythmogenic cardiac laminopathy. PLoS One. (2015) 10(4):e0121723. 10.1371/journal.pone.0121723 25837155 PMC4383583

[24] Brodehl A Holler S Gummert J Milting H. The N-terminal part of the 1A domain of desmin is a hot spot region for putative pathogenic DES mutations affecting filament assembly. Cells. (2022) 11(23):3906. 10.3390/cells11233906 36497166 PMC9738904

[25] Ferradini V Cosma J Romeo F De Masi C Murdocca M Spitalieri P Clinical features of LMNA-related cardiomyopathy in 18 patients and characterization of two novel variants. J Clin Med. (2021) 10(21):5075. 10.3390/jcm10215075 34768595 PMC8584896

[26] Bottigliero D Monaco I Santacroce R Casavecchia G Correale M Guastafierro F Novel AKAP9 mutation and long QT syndrome in a patient with torsades des pointes. J Interv Card Electrophysiol. (2019) 56(2):171–2. 10.1007/s10840-019-00606-y 31418098

[27] Algra A Tijssen JG Roelandt JR Pool J Lubsen J. QTc prolongation measured by standard 12-lead electrocar-diography is an independent risk factor for sudden death due to cardiac arrest. Circulation. (1991) 83(6):1888–94. 10.1161/01.CIR.83.6.1888 2040041

[28] Marcus FI Fontaine GH Frank R Gallagher JJ Reiter MJ. Long-term follow-up in patients with arrhythmogenic right ventricular disease. Eur Heart J. (1989) 10(suppl_D):68–73. 10.1093/eurheartj/10.suppl_D.68 2806306

