# Supplementary figures and images for: A case of arrhythmic cardiomyopathy caused by rare multiple gene mutations
Source: Front Cardiovasc Med. 2025 Jul 10;12:1598085. doi: 10.3389/fcvm.2025.1598085 (PMC12286949; doi:10.3389/fcvm.2025.1598085)

The Family Sanger Sequencing Results

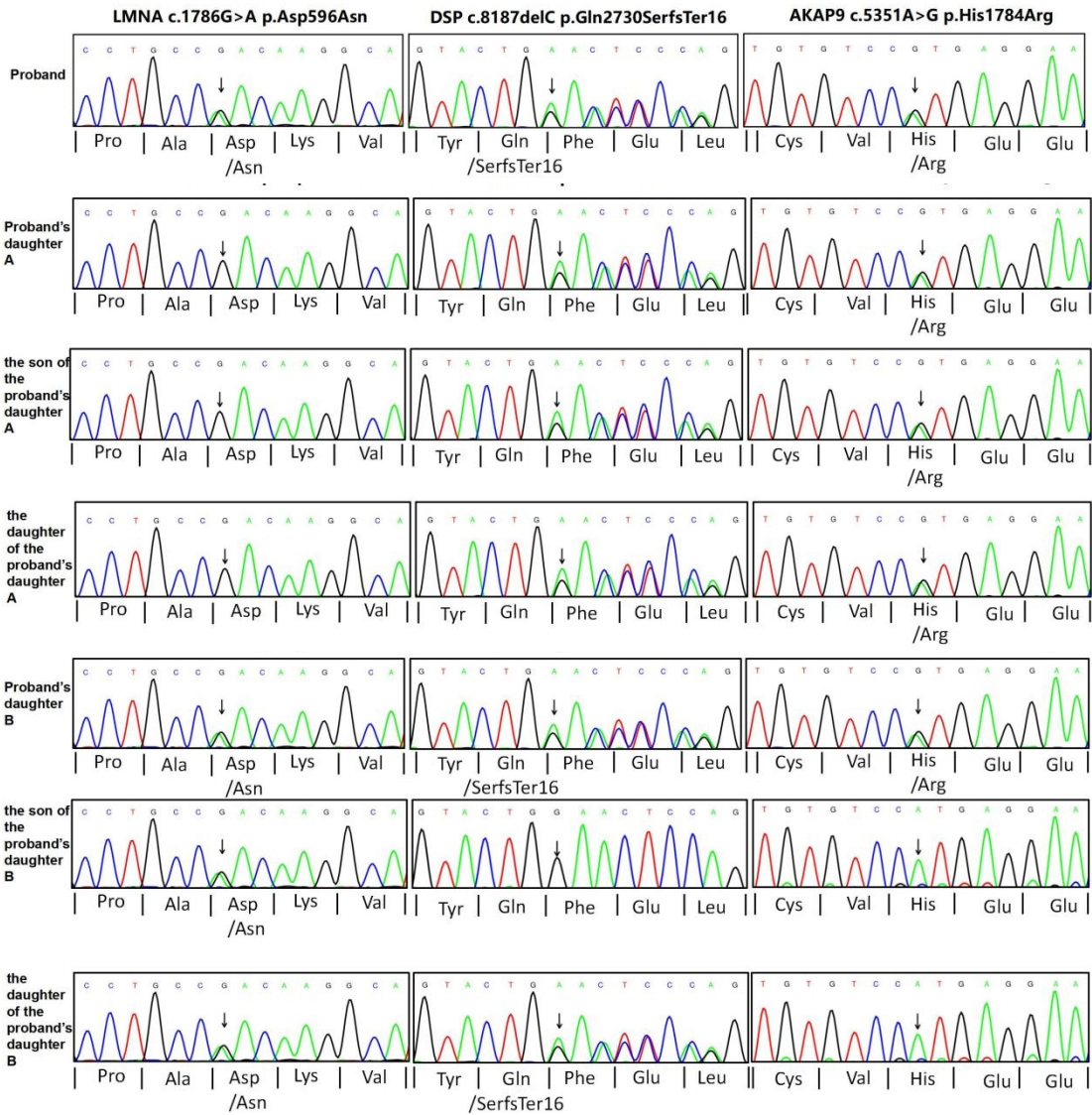

Supplement: Supplementary file 1 [file Image1.pdf]
