# Supplementary material for: A case of arrhythmic cardiomyopathy caused by rare multiple gene mutations
Source: Front Cardiovasc Med. 2025 Jul 10;12:1598085. doi: 10.3389/fcvm.2025.1598085 (PMC12286949; doi:10.3389/fcvm.2025.1598085)
Supplement: Supplementary file 2 [file Datasheet1.pdf]

Supplementary materials for the proband's CMR

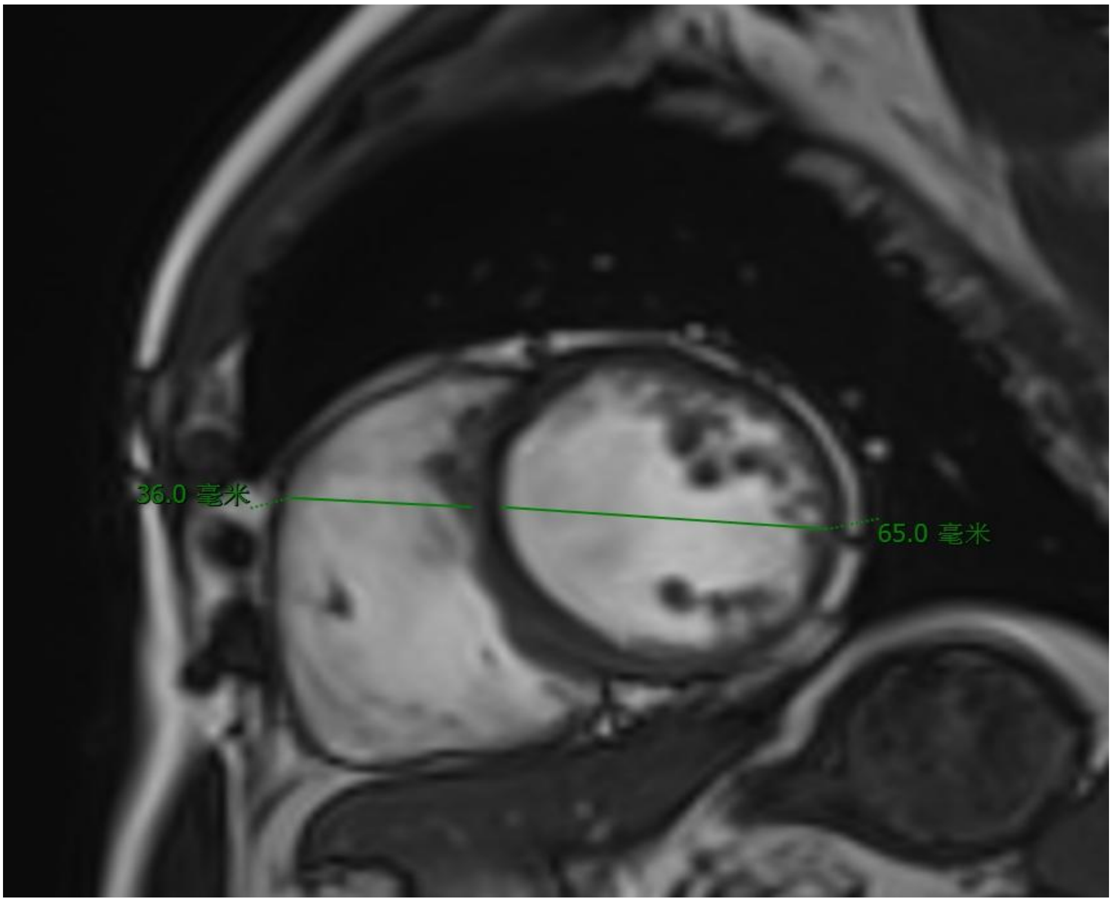

Figure 1. Short-axis cine MRI: Biventricular enlargement, right ventricular end-diastolic diameter 36 mm, left ventricular end-diastolic diameter 65 mm.

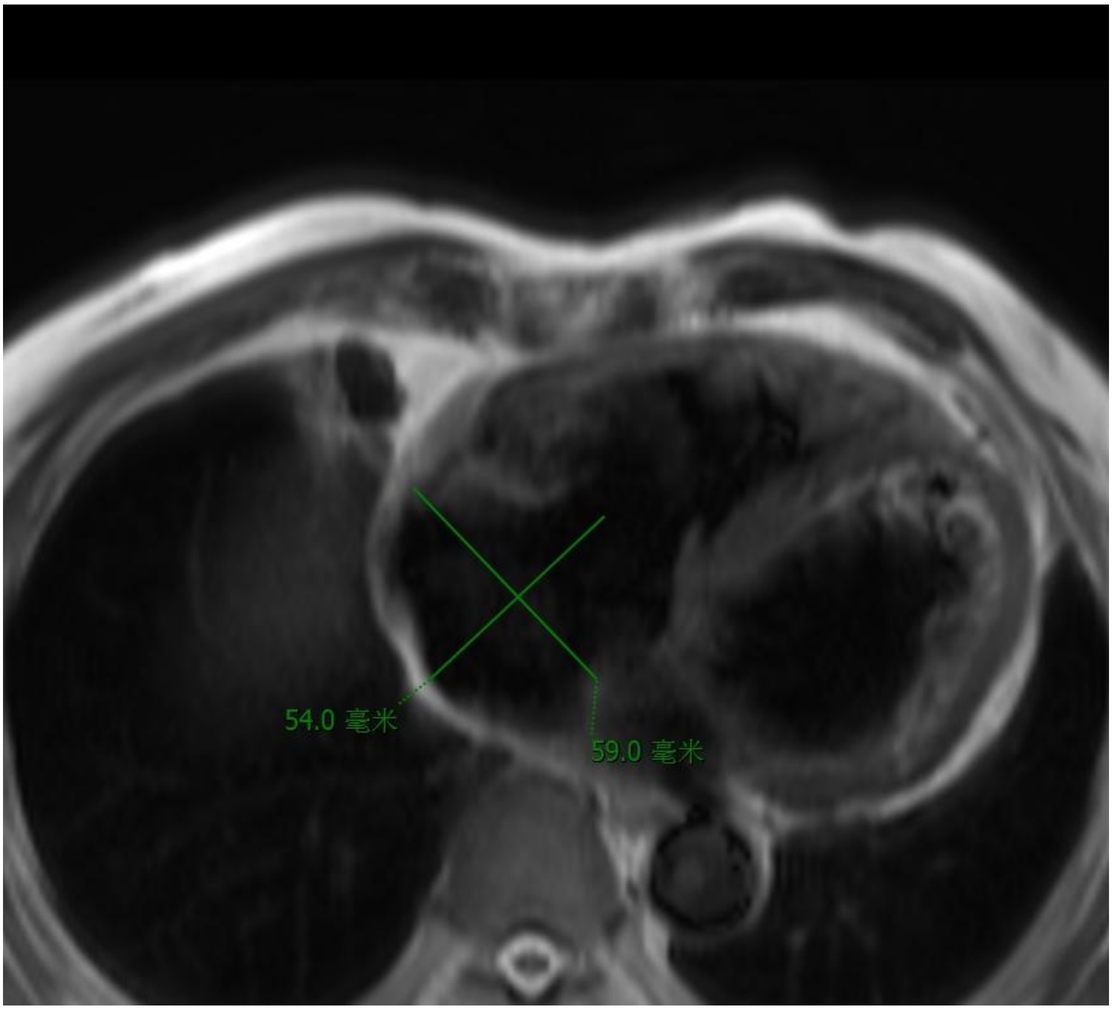

Figure 2. Axial HASTE: The right atrium is enlarged, with an anteroposterior diameter of 54 mm and a transverse diameter of 59 mm.

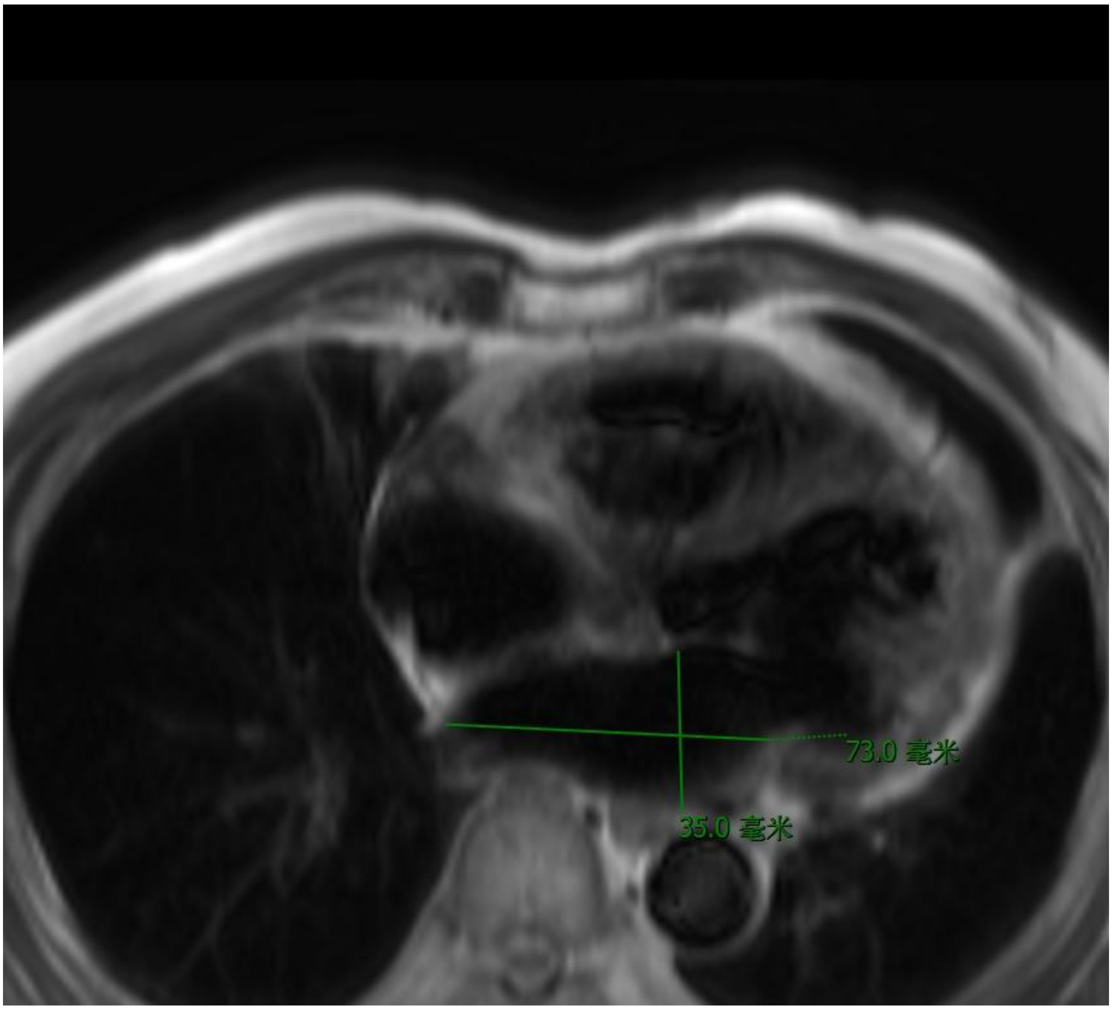

Figure 3. Axial HASTE: Left atrial enlargement, with an anteroposterior diameter of 35 mm and a transverse diameter of 73 mm.

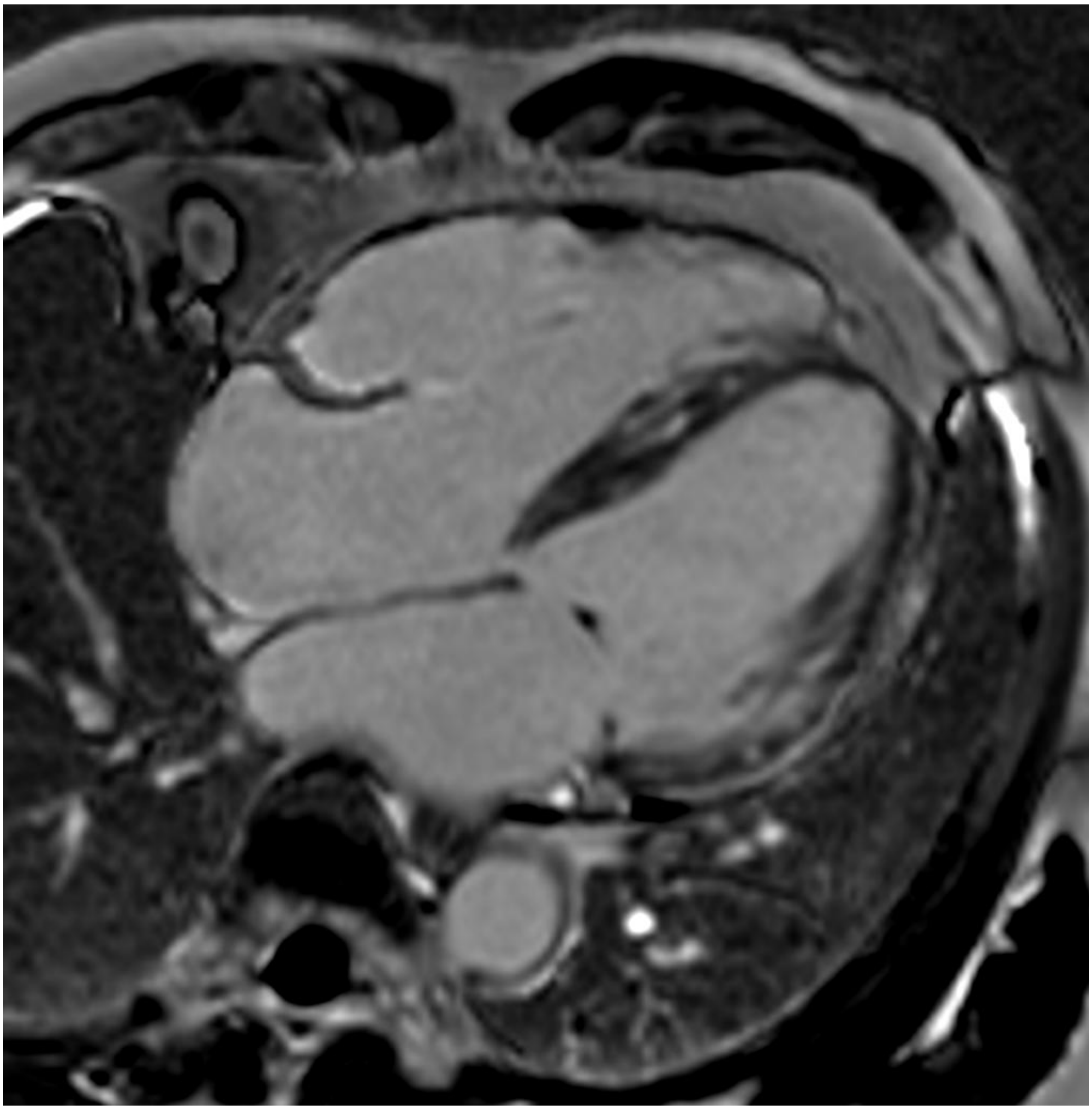

Figure 4. Four-chamber LGE: Epicardial fibrosis in the lateral wall of the left ventricle and intramural myocardial fibrosis in the interventricular septum.

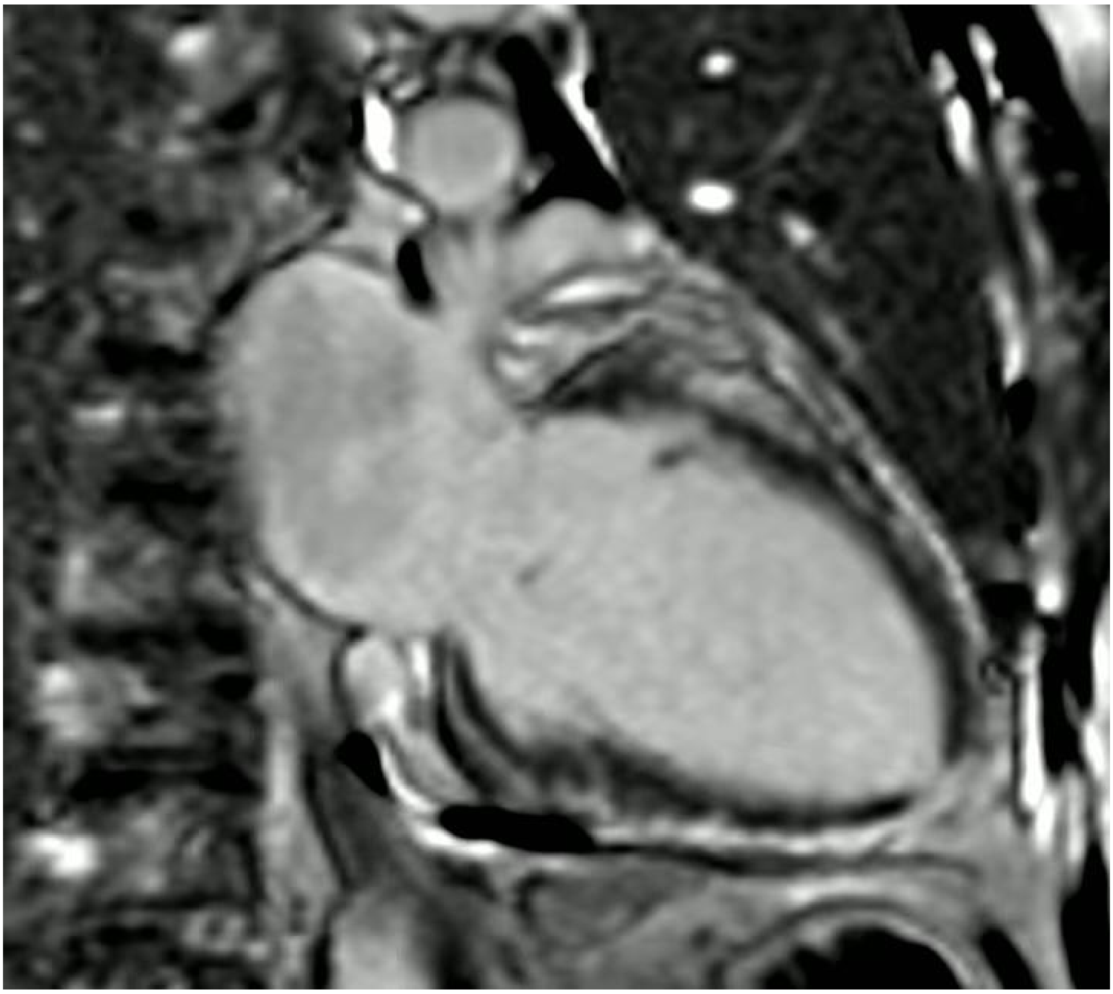

Figure 5. Two-chamber view LGE of the left ventricle: myocardial fibrosis in the subepicardium of the inferior wall and anterior wall of the left ventricle.

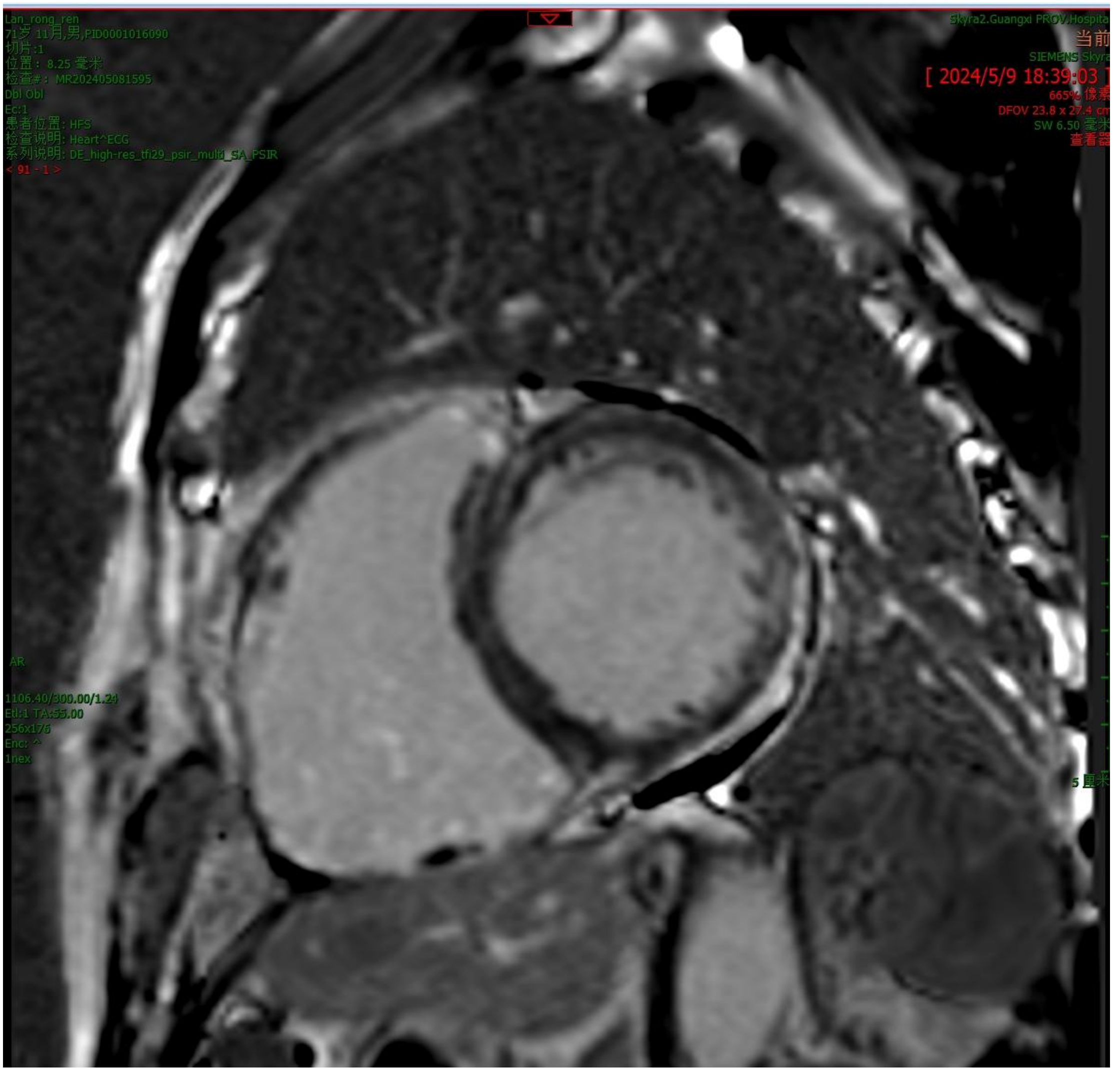

Figure 6. Short-axis LGE (basal segment): Subepicardial myocardial fibrosis in the lateral and inferior walls of the left ventricle, mid-myocardial fibrosis in the anterior wall and interventricular septum, and transmural myocardial fibrosis in the inferior wall of the right ventricle.

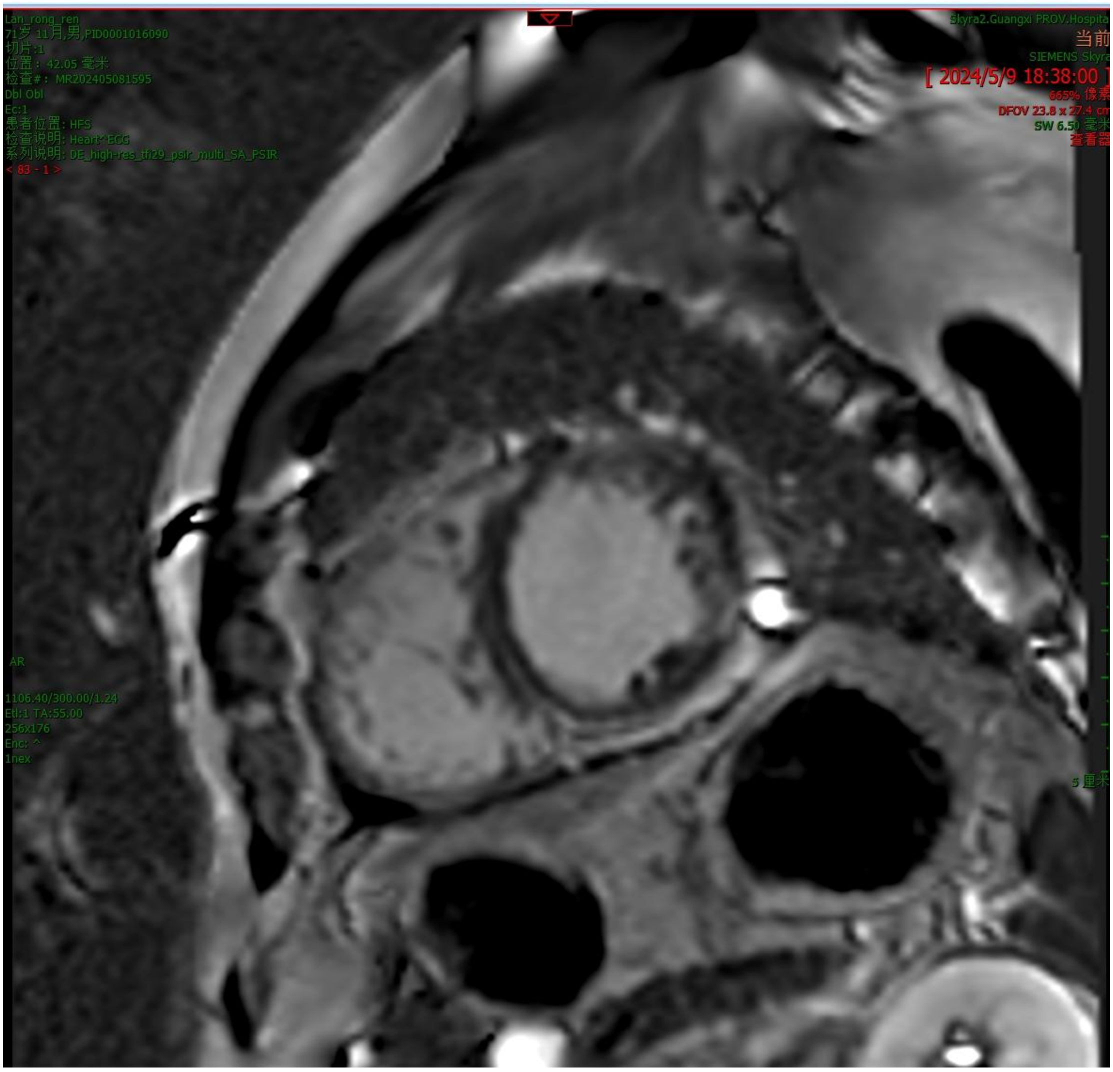

Figure 7. Short-axis LGE (distal segment): Fibrosis in the epicardial myocardium of the left ventricular lateral wall and inferior wall, as well as intramural myocardial fibrosis in the interventricular septum.
